# Supplementary material for: Impact of systematic medication review in emergency department on patients’ post-discharge outcomes—A randomized controlled clinical trial
Source: PLoS One. 2022 Sep 19;17(9):e0274907. doi: 10.1371/journal.pone.0274907 (PMC9484649; doi:10.1371/journal.pone.0274907)
Supplement: S2 Appendix — (PDF) [file pone.0274907.s002.pdf]

S2 Appendix

Contents

Original trial protocol	Page 2
Protocol amendments	Page 17
Timeline of the study, milestones	Page 18

Original trial protocol

Improving drug safety in emergency patients –a randomised controlled trial to investigate the effect of medication reconciliation and review on readmission rate

Study group:

Study leader: Erik H. Øie, dr.med, Chief physician medical department, Diakonhjemmet Hospital

Ph.D. –student and study manager: Lisbeth Damlien, MSc Pharmacy

Main supervisor: Kirsten K. Viktil, Associate professor II, University of Oslo, Head of Research and Pharmaceutical service. Diakonhjemmet Hospital Pharmacy

Co-supervisor: Espen Molden, Professor II, University of Oslo and Research leader, Center for Psychopharmacology, Diakonhjemmet Hosipital

Study collaborators: Ole K. Roald, dr.med., Head of the Anaesthesia and Intensive care department at Diakonhjemmet Hospital

Lasse Andreassen, MSc Health Management, unit manager at the emergency department, Diakonhjemmet Hospital

Statistician: Tron Anders Moger, PhD, MSc in Statistics, Department of Health Management and Health Economics, University of Oslo

Patient representative: Liv Hopen, member of the Norwegian Heart- and Lung Association

Reference group:

Liv Mathisen, Ph.D., Head of Research and development at Hospital Pharmacies Enterprise, South Eastern Norway

Marianne Lea, MSc Clinical Pharmacy, Ph.D.-student at Hospital Pharmacies Enterprise, South Eastern Norway

Ulrika Gillespie, PhD, Researcher at Uppsala University Hospital, Uppsala, Sweden

Summary

Background: An accurate medication history is a vital part of any hospital admission. However, studies have shown that 60-70% of hospitalised patients have at least one unintended medication discrepancy between their actual (‘home’) drug treatment and medication list registered at hospital admission. Medication discrepancies and drug-related problems (e.g. adverse drug reactions) is a recognised health care challenge. Currently there is lack of studies investigating the effect of interventions and actions to improve the quality of medication history recording and assessments in the emergency setting where critical decisions are made regarding further ‘patient processing’. **Objective:** To test if a new working model combining medication reconciliation and medication review in emergency patients can decrease the readmission rate. **Study design:** Randomised, controlled trial at the Emergency Department, Diakonhjemmet Hospital. The control group receives standard care, while in the intervention group a clinical pharmacist is integrated in the interdisciplinary team and conducts medication reconciliation and review.

1. Background

An accurate medication history is a vital part of any hospital admission. As different sources present different information about the patient's medication history, it can be challenging identifying which medications the patients actually have been using (1-3). Studies have shown that 60-70% of hospitalised patients have at least one unintended medication discrepancy regarding their home medication regimen and the admission orders (4-8). Further, studies have estimated that 15% of hospital admissions in elderly patients are cause by adverse drug events (drug-related problems) (9, 10), studies indicates that majority of these admissions could have been prevented (10-12). Admission to an emergency department is a key vulnerable moment when patients are at increased risk of medication discrepancies, and also identification of relevant adverse drug events, such as drug-related cause of admission, is crucial. If medication discrepancies and drug-related causes of admission are not revealed in the emergency department, physicians at hospital wards potentially can inflict the patients with side-effects, interactions or therapeutic failure.

High risk patients

Through a pilot study conducted in 2014 in the emergency department, Diakonhjemmet Hospital, we found that approximately 40% of patients were admitted to the emergency department regarding their heart or lung disease. About 60% of these patients had 3 or more registered diseases and thereby had a higher risk of having a clinical relevant medication discrepancy, according to our results. Co-morbidity and extensive use of medication have also been proven as risk factors for medication discrepancies and drug-related problems by other researchers (13-19). Data from Diakonhjemmet Hospital estimates that 29.2% of patients admitted to Diakonhjemmet Hospital with asthma/COPD related cause of admission were readmitted within 30 days. Also patients with heart failure are at high risk for readmissions; 24.3% of patients admitted to Diakonhjemmet Hospital with heart failure were readmitted within 30 days. In the impending study we will get an overview of what patients are at increased risk of drug-related admissions and drug-related problems at admission, but patients with heart or lung-diseases is two of our focus groups, due to the high proportion of co-morbidity and high risk of readmissions in these patients.

Medication reconciliation and medication review

Medication reconciliation is the systematic process of obtaining a complete overview of the patients medications, including name, strength, dosage and route of administration. Preferably this is obtained by interviewing the patient and using a checklist, when needed, complimentary information is obtained from relevant level of care. If the patient is not in charge of their medication an updated medication list is obtained from the relevant level of care. Medication review is the systematic process of evaluating the patient's medication regimen individually to optimise the effect of and reduce the risk of medication use.

Medication reconciliation performed at hospital wards within 48 hours after the patient is admitted, is proved through both national and international studies to be an effective way of reducing the number of medication discrepancies (20-22). However a recently report from the Norwegian knowledge centre for the health service states that there is lack of studies investigating the clinically relevant outcome of performing medication reconciliation, e.g. effect on readmissions and length of stay (22). To identify, prevent, and solve clinical relevant drug-related problems such as interaction, adverse drug reactions, too high dosages etc. a systematic medication review is shown to be an effective method (23, 24), traditionally this is conducted during the hospital stay. It is a fact that the length of stay in Norwegian hospitals is becoming shorter, and therefore, in a perspective of patient safety and also to secure an

effectively hospital stay, medication reconciliation and medication review could advantageously be conducted during the stay at the emergency department. This to ensure that the physician at the emergency department has all the information he need to make an informed decision about the patient being hospitalised or not, and about the further treatment of the patient.

In the earlier mentioned pilot study we developed a working model for conducting medication reconciliation at the point of admission, and further, we evolved a prioritising model for identifying patients with increased risk of medication discrepancies at admission to the emergency department (paper submitted). We found that 62% of the patients admitted to the emergency department had one or more clinical relevant medication discrepancy when we compared the medication list obtained by physicians in the emergency department and the list obtained through medication reconciliation. We also found that the working model we developed was perceived efficient by physicians at the emergency department. In Norway it is currently no established procedure for systematically conducting medication reconciliation and medication review at the point of admission to the emergency department. The clinically relevant outcome of conducting these interventions at the point of admission to the emergency department is scarcely investigated.

2. Hypothesis and objectives

2.1 Research hypothesis

Implementation of a working model for combined medication reconciliation and medication review at point of admission to the emergency department will improve drug safety and reduce the proportion of patients who are readmitted after 12 months (included visits to the emergency department).

2.2 Objective

The overall primary objective of this study is to test if a working model for performing medication reconciliation and medication review at the emergency department can decrease proportion of patients who is readmitted (included visits to emergency department).

Secondary objectives is to test if the working model for performing combined medication reconciliation and medication review at the emergency department can decrease the average length of stay in the emergency department and for the total hospital stay.

And also investigate if the working model can increases the proportion of patients who are sent home or is referred to the out-patient-clinic opposed to being hospitalised.

Further, it will be investigated if a prioritising model can be used to predict what patients have the highest risk of drug-related admissions and drug-related problems at admission to the emergency department.

We will be investigating if the new working model is perceived as effective by the health personnel and patients at the emergency department through a semi structural questionnaire.

To obtain the patients perspective of the challenges outlined in this study, we will invite a random sample of included patients to a group interview.

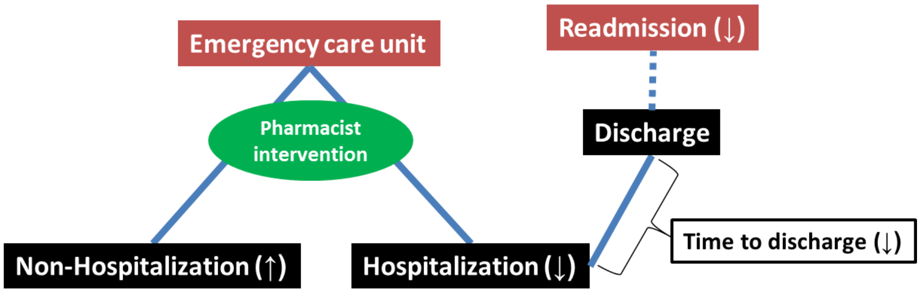

Figure 1: Illustration of the hypothesis of this study, i.e. that the intervention increases the proportion of non-hospitalised patients (indicated by upward arrow) while decreasing hospitalisation degree, readmission rate (primary endpoint) and time to discharge (indicated by downward arrows)

3. Methods

3.1 Study design

This is a randomised, controlled trial, non-blinded. Patients admitted to the emergency department will be included. Patients will be randomised into two groups; one control group, who will receive standard care and one intervention group, who will receive medication reconciliation and medication review at the emergency department. These interventions will be conducted by a clinical pharmacist in the interdisciplinary team. The study design is illustrated in figure 2.

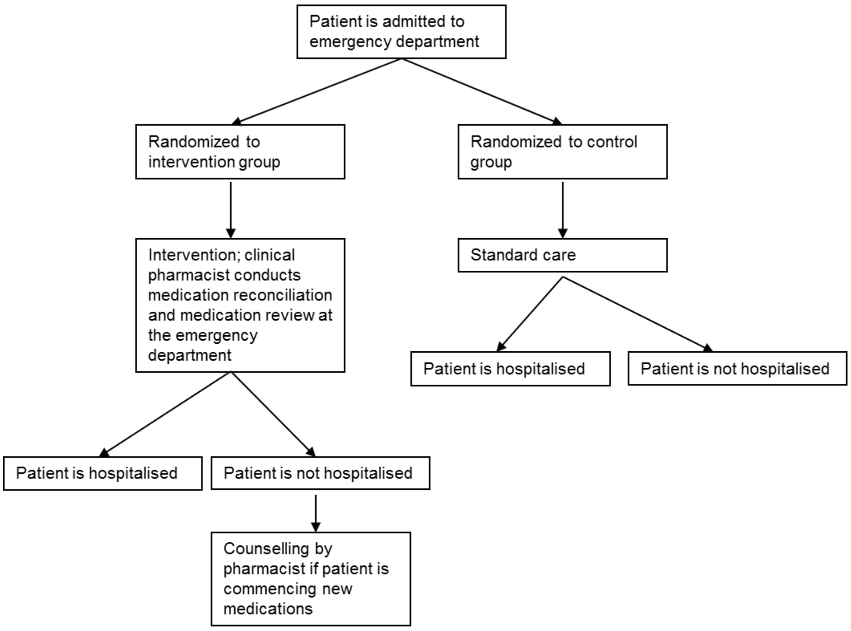

Figure 2: Illustration of the study design

3.2 Patients and study settings

Patients admitted to the emergency department at Diakonhjemmet Hospital will be included consecutively. Yearly about 13.000 patients are presented to this emergency department and the

number has increased rapidly over the past years. In 2015, 40-45 patients are daily presented to the emergency department. Patients with medical and surgical issues are admitted to the same emergency department and therefore both groups of patients will be included in the study. However, elderly patients with hip fractures are because of high risk of post-operative infections fast-tracked directly to a surgical ward and are not triaged or examined in the emergency department, hence these patients will not be included in this study. The mean length of stay at the emergency department at Diakonhjemmet Hospital is 2.8 hours (2014). The study will be carried out as collaboration between the emergency department at the hospital and the hospital pharmacy.

3.3 Inclusion and exclusion criteria

Patients will be included if they meet the following criteria:

- Patients ≥ 18 years admitted to the emergency department
- Able and willing to provide written consent (*see 3.4 Inclusion procedure and 3.13 Ethics*)

Patients will be excluded if they meet one of the following criteria:

- Patient are already included
- Terminal ill patients with short life expectancy
- Control group patients where physician at the emergency department request an assessment from a pharmacist
- Control group patients where the study pharmacist reveal drug-related problems of major clinical relevance and has to intervene

3.4 Inclusion procedure

Staff at the emergency department, including physicians and nurses will be informed about the study. At admission, if the patient is eligible, the study pharmacist will describe the study to each potential participant and/or their next of kin, then provide written information about the study and answer potential questions. If patients temporary are unable to consent when asked to participate (e.g. delirium) their next of kin will be asked to supply a preliminary consent in the patients place. If the patient later refuses to participate he/she will be excluded from the trial, and any registered data on the patient will be deleted.

3.5 Randomisation

Patients will be randomised to control- or intervention group at admission to the emergency department. We will randomise the days and not patients, this to reduce the spill over of methodology because the same nurses and physicians are involved in both study groups (*see 8. Risk management)*. Per week we therefore will randomise what days will be intervention days and what days will be control days. The randomisation process will be conducted by department of Biostatistics and epidemiology at Oslo University Hospital. They will deliver randomisation envelopes, and the study manager will follow randomisation procedure for all included patients.

3.6 Standard care

A physician and a nurse will perform relevant examinations when a patient is admitted to the emergency department, and further, it will be decided if the patient will be hospitalised or not. Pharmacist will not intervene on these patients. Before the patients are transferred to a specialised

ward, the physician will acquire the patient’s medication history, either by asking the patient and/or using available information from relevant sources for instance from the referral papers. This is the standard routine care as it is performed today. The medication list is documented in the electronic patient record and also handwritten by the physician in the medication chart.

3.7 The intervention

3.7.1 Admission

In addition to physicians, nurses and others, pharmacist will be a part of the interdisciplinary team at the emergency department. Medication reconciliation will be conducted by a clinical pharmacist before the patient’s medication history is registered by the physician at the emergency department. Information obtained will be communicated to the physician in charge of the patient. Medication review will be conducted on the basis of data from the medication reconciliation and available clinical information; clinical chemical information is available in most patients within short time of admission. Revealed drug-related problems relevant to the admission will be discussed with physician at the emergency department. Other drug-related problems considered clinical relevant by the study pharmacist will either be discussed with physician at the emergency department or be documented in the electronically patient journal for follow up at the hospital ward or general practitioner/nursing home etc. if the patient is not hospitalised.

3.7.2 Discharge from the emergency department:

When an intervention group patient is discharged directly from the emergency department, with new medications prescribed, an education session with the pharmacist will be arranged. The goal is that patients get all the information they need to use their medicines correctly after discharge. The patients are encouraged to ask questions about their medicines during this session. If any additional drug-related problems are identified during the education session, these will be discussed with the physician at the emergency department immediately, i.e. before the patient leaves the hospital.

3.8 Data collection and follow up

The data collection for the study will start 01.05.2016. A total of 800 study participants will be included (*see 3 .11 Sample size calculation*).

Baseline data will be collected at inclusion for both study arms. Data will be collected from hospital and pharmacy records, general practitioners, primary care (e.g. nursing home, community health service), patients and/or relatives. General demographics to be collected include age, gender, cause of admission to the emergency department, help from community health services with medications and delivery of multi-dosage packed medications, earlier registered medication history. For the control group medical and medication history will be obtained from the electronically patient journal and medication charts. For the intervention group, information will be collected as described in the medication reconciliation procedure above. If other clinical pharmacists at the different hospital wards intervene on the patients during hospital stay, this will be registered. Follow-up regarding registration of readmissions will be recorded at 6 and 12 months after inclusion for both study arms. To be able to register readmissions access to the Norwegian Patient Registry must be granted.

Clinical relevance of the drug-related admissions and other drug-related problems revealed will be evaluated retrospectively by an interdisciplinary team, using a published scale (25).

3.9 Data management

Each study participant will be given a unique study number. The code list will be kept electronically in the hospitals password-secured research server. Patient data will be collected on a customised data collection form; this form will be piloted during the study preparation phase (*see 4. Progress plan*). Patient-identifiable data registered on paper forms will be stored at the hospital in accordance with hospital journal information routines (*see 3.13 Ethics*).

3.10 Outcome measures

Primary endpoint:

- Difference between control and intervention groups in proportion of patients readmitted to any hospital within 12 months (endpoint including revisits to the emergency department)

Secondary endpoints:

- Difference between control and intervention groups in proportion of patients not hospitalised following admission to in the emergency department
- Difference between control and intervention groups in the length of stay at the emergency department.
- Difference between control group and groups in the overall length of hospital stay
- Describe the frequency of drug-related admissions in the intervention group, describe consequences, out-come and follow up for these patients.
- Difference between control group and intervention group, in regards to average time to next contact with hospital and average number of readmissions.

Other outcomes:

- Describe workflow, information flow and multidisciplinary collaboration using results from survey amongst the involved physicians and other healthcare personnel at the emergency department and relevant hospital wards
- Describe patients view on medication regimen, believes and concerns about medication (26, 27), medication lists and drug-related admissions using results from group interview and survey amongst a randomised sample of patients. Every 10th included patient (10%) will retrospectively be invited to participate in the group interview. And every 4th included patient (25%) will retrospectively be invited to fill out a survey.

3.11 Sample size calculation

Available information about readmission frequency at Diakonhjemmet Hospital is based on 30 days follow up, and therefore cannot be used to calculate proportion of patients readmitted after 12 months. However, numbers from Oslo University Hospital estimate a readmission proportion of 50% after 12 months in a comparable patient population. We therefore use this estimate as the expected readmission rate in the control group.

In a previous Swedish study conducted by Ulrika Gillespie (12) who is member of our reference group, a 16% reduction in hospital revisits within 12 months was found amongst older patients (>80 years) following a comparable intervention as described in our protocol.

On this basis, it will be necessary to include at least 146 patients in each group to show a significant effect on the primary endpoint (significance level of 5%, study power of 80%). However, the elderly patients included in the Swedish study have more comorbidity and therefore more use of health care resources. The patients in our study will be 18 years and older and thereby the difference between our

control group and intervention group probably will be smaller. A more realistic difference between our groups is 10%, thereby 385 patients would have to be included in each group to show a significant effect on the primary endpoint. To compensate for dropout we aim to include 400 patients in each study group, thus a total of 800 patients. Based on statistics from Diakonhjemmet Hospital, inclusion of this amount of patients from the Emergency Department would require an inclusion period of 12 months.

3.12 Statistics and analysis

Statistical analyses will be conducted in IBM SPSS Statistics. Data will be assessed for normality and analysed according to appropriate statistical tests. The baseline demographic and clinical characteristics will be summarised using proportions, means and standard deviations, or median and interquartile range, as appropriate. Baseline comparisons: characteristics of study participants in the two study groups will be compared using the chi-square test for categorical variables and the Student's *t*-test or non-parametric equivalent (e.g. the Mann-Whitney *U* test) for continuous variables. Multivariable analysis (logistic regression) will be used to compare endpoints between study groups while adjusting for prognostic variables and potential confounders. All statistical tests will be interpreted with a significance level of 5% (two-tailed). For building the model for prioritising patients at increased risk of drug-related admissions and drug-related problems at admission to the emergency department binary regression analysis will be used. Data will be analysed according to intention-to-treat (ITT) principles. In addition to ITT analysis, per protocol analysis will also be performed.

3.13 Ethics

Implementing a working model for medication reconciliation and medication review in the emergency department will not have any other disadvantages for the patients than they may, in the study setting, have to answer the same questions several times and this may be an additional burden. Overall the patient will probably benefit from participating in the study, as their medication list will be quality assured, and assessed for drug-related problems at admission to the emergency department. The study will however strive for establishing a working model causing the patient least possible burden. The procedures implemented in this study will not delay the acute treatment of the patient.

Preferably patients will be asked for written consent before they are included in the study. Although in the acute situation many patient will temporarily not be able to give written consent for participating. However it is not ethical just to exclude these patients since our hypothesis is that medication reconciliation and medication review are beneficial for the patients. In such cases the patient will be asked for written consent as soon as he or she is able to do so or their next of kin will be asked to supply consent in the patients place. If the patient later refuses to participate he/she will be excluded from the trial and all registered patient data will be deleted. Patients who are mentally unable to consent to participate, their next of kin will be asked to supply consent in the patients place.

Patient data will be registered on paper forms, which will be de-identified after the patient data is transferred de-identified to the study database on the hospital research server; password protected. Only a code list will connect the patient to his or her data. Paper forms will at all times be kept locked in a fire safe cabinet and be accessible only to authorised study personnel, and eventually the forms will be maculated. De-identified patient information will not be brought out of the hospital. The code list connecting the patients to their data will at the latest be deleted 3 years after start of data collection.

When results are published it will not be possible to identify individual patients.

An application for ethical approval will be submitted to the Regional committee for medical and health research ethics (REC). The study protocol also has to be approved by the research committee at Diakonhjemmet Hospital.

4. Progress plan

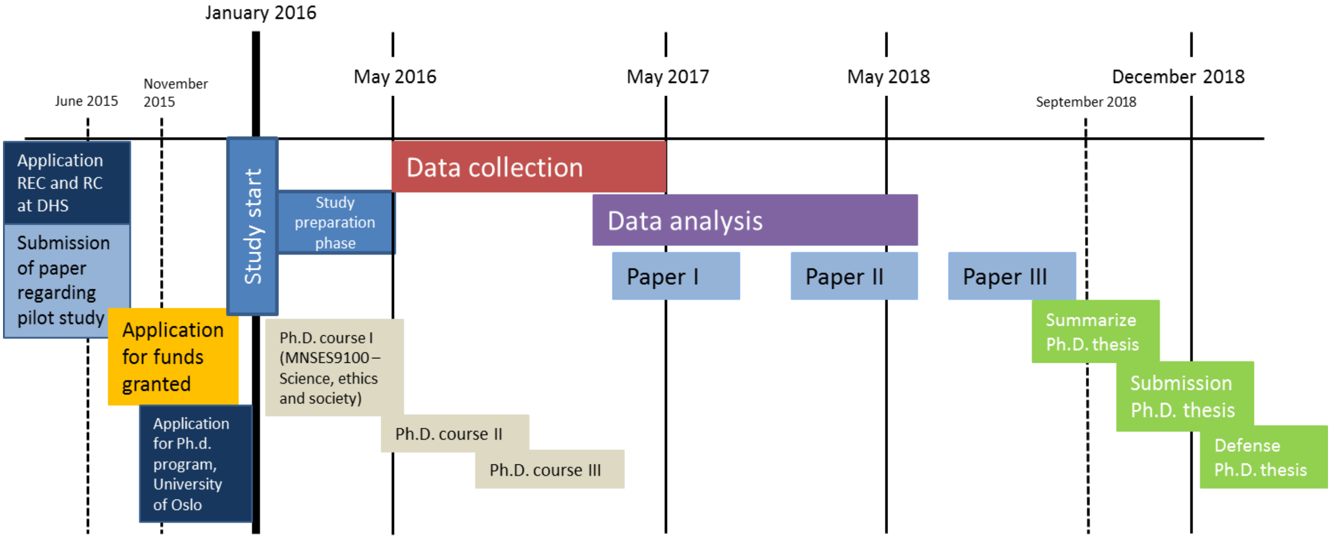

Illustration of progress plan, REC: Regional committee for medical and health research ethics, RC at DHS: the Research Committee at Diakonhjemmet Hospital.

- Before study start at 1. January 2016, an application for ethical approval will be submitted to the Regional Committee for Medical and Health Research Ethics (REC). The study protocol will also be sent to the Research Committee at Diakonhjemmet Hospital (RC at DHS) for approval.
- The Ph.D.-Candidate will apply for admission to the Ph.D.-program at the University of Oslo before study start. The Ph.D.-program require that the Ph.D. –candidate complete Ph.D.-courses rewarded with at least 30 credits, the course MNSES9100 –Science, ethics and society is mandatory.
- In the study preparation phase from 01.01.16-01.05.16 preparations for the data collection is done (pilot-test the data collection forms, inform the staff at the emergency department, prepare the study-database etc.)
- Data collection starts 01.05.16 and will continue for approximately one year or until 800 patients is included.
- 2017-18: analysing data and preparing/organising data for publication of papers
- 2017: Publication first paper
- 2018: Publication second paper
- 2018: Publication third paper
- 2018: Summarize Ph.D.-thesis
- December 2018: Submission Ph.D.-thesis
- January 2019: Defence of the Ph.D.-thesis

5. Patient involvement

A patient-representative has been involved in the evolvement of the study design. The patient-representative will be involved in evaluation of the working model for medication reconciliation and medication review in the emergency department. When the results of the study are disseminated the patient-representative will be important for distributing the results to patient organisations. The patient perspective is important in the clinical practice, therefore a survey regarding the effectiveness of the new working model in the emergency department will be conducted amongst a randomised sample of the included patients. The results of this survey will be part of the evaluation of the working model.

To get more insight in the patient's perspective of the challenges outlined in this study we will invite a random sample of included patients to a group interview. The results of the interviews will provide the basis for customising the working model to the patients' beliefs and needs.

6. Publication and dissemination of results

The already performed pilot study and planned RCT will provide data for at least four scientific papers (see specifications below), which will have the potential for publications in international peer-reviewed medical, pharmaceutical and nursing journals. We will aim for publications in recognized journals, and pay for "open access".

1. Drug Safety at admission to Emergency Department - an innovative model for prioritising patients for Medication Reconciliation (PRIOMER) (Submitted)
2. Drug safety at admission to emergency department -an innovative model for prioritising patients for medication review
3. A novel interdisciplinary model at an emergency department –how does it influence readmission rate and how does it influence work flow and effectiveness?
4. Incidence of drug-related admissions to a Norwegian emergency department –could some of the admission have been prevented?

During the study period, the Ph.D.-student will attend and present research results for instance at the following meetings:

- The European Society for Emergency Medicine
- International Conference of Emergency Medicine
- The International Society for Pharmacoepidemiology Congress (ISPE)
- European Society of Clinical Pharmacy (ESCP)
- Nordic Social Pharmacy and Health Services Research Conference
- Norwegian yearly conference on patient safety

A key component in this study is also to disseminate the results to the relevant patients groups. We will take advantage of the hospitals own Department of Communication, which helps researchers with dissemination of results. Additionally dissemination channels of the Norwegian Heart- and Lung Association, our collaborator will be utilized to disclose the results from the study. And also the patient representative will be involved in the dissemination process.

7. Foundation of the study

The study is a collaboration between the emergency department, Diakonhjemmet Hospital and Diakonhjemmet Hospital Pharmacy, head of both divisions supports the study.

Since 2011 the Norwegian patient safety program, initiated by the Norwegian Ministry of Health and Care Service, has been focusing on medication reconciliation conducted in hospitals as one of several initiatives to reduce patient harm. According to this Diakonhjemmet Hospital has implemented some of the initiatives presented by the patient safety program to reduce medication discrepancies in hospital. Our study does not inflict with the initiative in the Norwegian patient safety program, on the contrary our study will give additional information about how to perform medication reconciliation in the most efficient manner in the hospital setting and also information about clinically relevant outcomes of medication reconciliation. The common focus on medication reconciliation indicates that the challenges outlined in our study are challenges that also are identified by the Norwegian authorities.

8***. Risk management***

The most important risk of the study is not to reach the needed number of patients to get enough power to receive statistical significant results. If we after 6 months have not recruited 50 % of the patients we will recruit more clinical pharmacists to include patients. Another present risk is the risk of spill over of methodology because the same nurses and physicians are involved in treating patients in both study groups. However, we believe that the intervention, the methodology of medication reconciliation and medication review, is so comprehensive that it is not easily transferred without being thoroughly taught and trained. We also will randomise the days for intervention and control to try to control the spill over effect.

As part of the hospital's aim of improving medication safety, some of the elements of the medication reconciliation might be implemented as part of standard care during the study period (*see 7. Foundation of the study)*. This might reduce the differences between the groups. We can only handle this by keeping track of patients and take this into concern in analysis.

At Diakonhjemmet Hospital clinical pharmacists is member of the interdisciplinary team at the hospital wards. Therefore patients in both study arms can be seen by a pharmacist during the hospital stay. This can affect the outcome in reducing the differences between the groups. We can only handle this by keeping track of patients and take this into concern in analysis.

References

1. Midlov P, Bergkvist A, Bondesson A, Eriksson T, Hoglund P. Medication errors when transferring elderly patients between primary health care and hospital care. Pharm World Sci. 2005;27(2):116-20.
2. Bakken K, Larsen E, Lindberg PC, Rygh E, Hjortdahl P. [Insufficient communication and information regarding patient medication in the primary healthcare]. Tidsskr Nor Laegeforen. 2007;127(13):1766-9.
3. Galvin M, Jago-Byrne MC, Fitzsimons M, Grimes T. Clinical pharmacist's contribution to medication reconciliation on admission to hospital in Ireland. Int J Clin Pharm. 2013;35(1):14-21.
4. Vira T, Colquhoun M, Etchells E. Reconcilable differences: Correcting medication errors at hospital admission and discharge. Quality and Safety in Health Care. 2006;15(2):122-6.
5. Rognstad S, Straand J. [Do general practitioners know what medication community nurses give their shared patients?]. Tidsskr Nor Laegeforen. 2004;124(6):810-2.
6. Myhr R, Kimsas A. [Medication errors when transferring within health care services]. Tidsskr Nor Laegeforen. 1999;119(8):1087-91.

7. Hellstrom LM, Bondesson A, Hoglund P, Eriksson T. Errors in medication history at hospital admission: Prevalence and predicting factors. BMC Clin Pharmacol. 2012;12(9).
8. Scullin C, Scott MG, Hogg A, McElnay JC. An innovative approach to integrated medicines management. J Eval Clin Pract. 2007;13(5):781-8.
9. Kongkaew C, Noyce PR, Ashcroft DM. Hospital admissions associated with adverse drug reactions: a systematic review of prospective observational studies. Ann Pharmacother. 2008;42(7):1017-25.
10. Beijer HJ, de Blaey CJ. Hospitalisations caused by adverse drug reactions (ADR): a meta-analysis of observational studies. Pharm World Sci. 2002;24(2):46-54.
11. Howard RL, Avery AJ, Howard PD, Partridge M. Investigation into the reasons for preventable drug related admissions to a medical admissions unit: observational study. Quality & safety in health care. 2003;12(4):280-5.
12. Gillespie U, Alassaad A, Henrohn D, Garmo H, Hammarlund-Udenaes M, Toss H, et al. A comprehensive pharmacist intervention to reduce morbidity in patients 80 years or older: A randomized controlled trial. Arch Intern Med. 2009;169(9):894-900.
13. Unroe KT, Pfeiffenberger T, Pharm SR, Jastrzembski J, Lokhnygina Y, Colon-Emeric C. Inpatient medication reconciliation at admission and discharge: A retrospective cohort study of age and other risk factors for medication discrepancies. American Journal Geriatric Pharmacotherapy. 2010;8(2):115-26.
14. Nester TM, Hale LS. Effectiveness of a pharmacist-acquired medication history in promoting patient safety. Am J Health Syst Pharm. 2002;59(22):2221-5.
15. Kramer JS, Hopkins PJ, Rosendale JC, Garrelts JC, Hale LS, Nester TM, et al. Implementation of an electronic system for medication reconciliation. Am J Health Syst Pharm. 2007;64(4):404-22.
16. Crook M, Ajdukovic M, Angley C, Soulsby N, Doecke C, Stupans I, et al. Eliciting comprehensive medication histories in the emergency department: The role of the pharmacist. Pharm Pract (Granada). 2007;5(2):78-84.
17. Walker PC, Bernstein SJ, Tucker Jones JN, Piersma J, Kim HW, Regal RE, et al. Impact of a pharmacist-facilitated hospital discharge program: A quasi-experimental study. Arch Intern Med. 2009;169(21):2003-10.
18. Forster AJ, Murff HJ, Peterson JF, Gandhi TK, Bates DW. Adverse drug events occurring following hospital discharge. J Gen Intern Med. 2005;20(4):317-23.
19. Field TS, Gurwitz JH, Harrold LR, Rothschild J, DeBellis KR, Seger AC, et al. Risk factors for adverse drug events among older adults in the ambulatory setting. J Am Geriatr Soc. 2004;52(8):1349-54.
20. Becerra-Camargo J, Martinez-Martinez F, Garcia-Jimenez E. A multicentre, double-blind, randomised, controlled, parallel-group study of the effectiveness of a pharmacist-acquired medication history in an emergency department. BMC Health Serv Res. 2013;13:337.
21. The Norwegian National Patient Safety Program; In safe hands, medication reconciliation: Programme secreteriate at the Norwegian Knowledge Centre for the Health Service and The Norwegian Directorate of Health; 2010. Available from: www.pasientsikkerhetsprogrammet.no.
22. Holte HH, Hafstad E, Vist GE Overview of reviews on effect of medication reconciliation: Nasjonalt Kunnskapssenter for helsetjenesten; 2015.
23. Blix HS, Viktil KK, Moger TA, Reikvam A. Characteristics of drug-related problems discussed by hospital pharmacists in multidisciplinary teams. Pharm World Sci. 2006;28(3):152-8.
24. Viktil KK, Blix HS. The impact of clinical pharmacists on drug-related problems and clinical outcomes. Basic Clin Pharmacol Toxicol. 2008;102(3):275-80.

25. Blix HS, Viktil KK, Reikvam A, Moger TA, Hjemaas BJ, Pretsch P, et al. The majority of hospitalised patients have drug-related problems: results from a prospective study in general hospitals. Eur J Clin Pharmacol. 2004;60(9):651-8.
26. Horne R, Weinman J, Hankins M. The beliefs about medicines questionnaire: the development and evaluation of a new method for assessing the cognitive representation of medication. Psychol Health. 1999(14):1–24.
27. Jonsdottir H, Friis S, Horne R, Pettersen KI, Reikvam A, Andreassen OA. Beliefs about medications: measurement and relationship to adherence in patients with severe mental disorders. Acta Psychiatr Scand. 2009;119(1):78-84.

Protocol amendments

September 9, 2016

- In the original trial protocol, it was proposed that written consent could be obtained from patients post-inclusion or from next of kin if the patient were not capable of providing this themselves during the emergency department stay. This was not approved by the Regional Committee for Medical and Health Research Ethics. A change in trial protocol were required for ethical approval.
  The inclusion criteria for the trial were changed to: *all patients arriving at the investigated emergency department, willing to/capable of providing written, informed consent.*
- Lasse Andreassen left the position as unit manager at the emergency department, Diakonhjemmet Hospital. Tord Kjesbu which were the new manager replaced Lasse Andreassen in the study group.

The change was approved by the Regional Committee for Medical and Health Research Ethics September 21, 2016.

March 14, 2017

After consultation with a statistician at department of Biostatistics and epidemiology at Oslo University Hospital, randomization procedure was altered. In the original protocol it was planned to randomise the data collection days and not patients, to reduce the spill over of methodology. However, the risk of selection bias when including intervention group patients and control group patients on different days, was considered higher than the risk of spill over of methodology. Hence, it was decided to randomize each patient at inclusion as described in the article.

March 17, 2017

- The start- and endpoint of the trial were changed, due to delay in financial support. Start of the trial was set to January 1, 2017, and endpoint March 31, 2021.
- In addition, a change in the original written consent scheme was necessary, this to clarify to participant what information the trial aimed to record and report.

The change was approved by the Regional Committee for Medical and Health Research Ethics March 21, 2017.

February-April 2017

Standard operation procedures were developed for:

- patient inclusion and randomization
- trial medication reconciliation and medication review
- registration of collected patient data

May 2017

The original protocol stated an exclusion criterion regarding terminal ill patients with short life expectancy. Sufficient information to clearly define these patients was not accessible in the fast-paced workflow in the emergency department. Hence, in May 2017 it was decided to include patients regardless of this exclusion criterion.

<u>June 8, 2018</u>

- A clarification regarding variables which should be harvested from the Norwegian Patient Registers was added to the protocol. The list of specified variables is listed below.

The clarification was approved by the Regional Committee for Medical and Health Research Ethics June 27, 2018.

List of specific variables harvested from the Norwegian Patient Registers approved by the Regional Committee for Medical and Health Research Ethics:

-Inndato og -tid for oppholdet (innDato / innTid) *(admission date and time)*
-Utskrivningsdato og –tid for oppholdet (utDato / utTid) *(discharge date and time)*
-Institusjonsnummer (org.nr) (institusjonID) *(institutional identification number of where patient was admitted)*
-Hvor pasienten kommer fra/ går til (fraSted / tilSted) *(indicator of where patients were admitted from and dicharged to)*
-Institusjonsnummer pasienten kommer fra/ går til (fraInstitusjonID / tilInstitusjonID) (*institutional identification number of what institution patient were admitted from and dicharged to)*
-Døgnopphold, dagopphold eller poliklinisk konsultasjon (omsorgsniva) *(indicator of the admission were an over-night stay, not an over-night stay or out-patient clinic contact)*
-Tidspunkt mht utskrivningsklar *(data when the patient treatment were completed during hospital stay)*
-Oppholdets liggetid *(length of stay)*
-Type kontakt, for polikliniske konsultasjoner/ dagbehandlinger (kontaktType)
-Klassifikasjonen av sykdommer og beslektede helseproblemer (ICD-10 diagnosekoder) *(ICD-10 code registered at discharge)*
-Angir om tilstanden er diagnostisert tidligere (nyTilstand) *(if the registered ICD-10 code was an earlier diagnosed condition)*
-Om oppholdet er et avdelings- eller sykehusopphold (niva) *(department or hospital stay)*
-Diagnoserelaterte grupper (drg) *(indicator of what condition were treated, used for economic analysis)*
-Korrigert vekt for drg poeng (korrvekt) *(indicator of what condition were treated, used for economic analysis)*
-Vekting av drg poeng (vekt) *(indicator of what condition were treated, used for economic analysis)*
-Antall liggedager innenfor aktuell DRG som er grunnlag for kostnadsvektberegninger
(trimpkt) *(indicator of length of stay for each treated condition, used for economic analysis)*
-Om DRG-en er medisinsk eller kirurgisk (M/K/blank) (drg_type) *(medical or surgical condition treated)*
-Kompliserende DRG (Ja/Nei) (komp_drg) *(complicating condisions)*
-Dagkirurgisk DRG (Ja/Nei) (dag_kir) *(surgical out-patient clinic conditions)*
-Spesifikk DRG (blank eller Ja) (spes_drg)
-Type rehabilitering (Vanling, komples eller sekundær) (rehabType) *(if admission could be classified as rehabilitation)*
-Gruppering av DRG-er til hoveddiagnosegruppe (hdg)
-Samtykkekompetanse *(if patient were competent of giving consent)*
-Informasjon om død og dødstidspunkt: død per 30. juni 2019? Dato for dødstidspunkt, dersom
død per juni 2019 *(information regarding death during follow-up)*

<u>August 2018</u>

The original protocol stated additional investigations of the study population and other investigations:

- Every 10th included patient (10%), and every 4th included patient (25%) retrospectively would be invited to participate in a group interview or fill out a survey, respectively. This to describe patients view on medication regimen, believes and concerns about medication (26, 27), medication lists and drug-related admissions.
- A survey amongst the involved physicians and other healthcare personnel at the emergency department and relevant hospital wards, should be conducted to describe workflow, information flow and multidisciplinary collaboration

Due to restricted resources, we were not able to perform these parts of the trial.
Workflow, information flow, and multidisciplinary collaboration was instead illustrated by implementation of pharmacists' recommendations by physicians.

<u>March 2020</u>

Data available from the Norwegian Patient Registry is routinely reported from all Norwegian hospitals. When receiving the data from the Norwegian Patient Registry it was revealed that how ED visits are reported vary between hospitals. Some hospitals report ED visits as a part of the hospital stay if patients are admitted, and outpatient clinic visits if patients are directly discharge from ED. Other hospitals strictly report ED visits as acute outpatient clinic visits. Originally, we planned to analyze the primary outcome divided into proportion of patient with an unplanned ED visit and proportion of patients with an unplanned readmission. However, the variety regarding coding of the ED visits in the NPR data, led to an uncertainty in the isolated ED-visit data. It was therefore decided to merge ED-revisits and hospital readmission in "unplanned contact with hospital" in analysis of outcome measures.

It was decided to add amendments in secondary outcome due to the relatively short intervention compared with the long follow-up time. The following secondary outcomes were added:

- Proportion of patients with an unplanned contact with hospital:
  - within 90 days after inclusion stay discharge.
  - within 30 days after inclusion stay discharge.

Timeline of the trial with milestones

May 2015: Original trial protocol written

June 2015-June 2016: Maternity leave PhD student

September 10, 2015: Ethical approval of the original trial protocol. The Regional Committee for Medical and Health Research Ethics (REC) approved the trial protocol. The trial was also approved by the Research Committee at Diakonhjemmet Hospital (September 2016).

January 1, 2017, to April 24, 2017: Pre-study period, practical planning of data inclusion period

April 2017: Registration and publication of the trial on clinicaltrials.gov`s website, based on the original trial protocol, Identifier: NCT03123640

April 24, 2017: patient inclusion started

May 16, 2018: patient inclusion completed

May 30, 2019: last day of follow-up on post-discharge outcomes

June 4, 2018: Application for harvesting outcome data was sent to the Norwegian Patient Registers**

April 2019 to February 2020: Maternity leave PhD student

January 15, 2020: Outcome data from the Norwegian Patient Registry was received

March 2020 to February 2021: Demographic data files prepared for analysis

February 2021 to June 2021: Outcome data files from Norwegian Patient registries prepared for analysis

August 2021 to November 2021: Outcome analyses conducted

**Huge workload at the Registers entails a very long processing time for outcome data.
