# Supplementary material for: Impact of systematic medication review in emergency department on patients’ post-discharge outcomes—A randomized controlled clinical trial
Source: PLoS One. 2022 Sep 19;17(9):e0274907. doi: 10.1371/journal.pone.0274907 (PMC9484649; doi:10.1371/journal.pone.0274907)
Supplement: S3 Appendix — (PDF) [file pone.0274907.s003.pdf]

S3 Appendix Detailed description of the drug-related problems categories

Table A. Description of drug-related problem categories. All drugs documented in the patients' reconciled drug lists were assessed according to these categories during medication review. The utilized drug-related problem categorization is based on a validated medication review-tool[1].

Drug-related problems categories	Detailed description
**Drug monitoring**	Therapeutic drug monitoring or laboratory monitoring was needed, for example for digoxin, warfarin, levothyroxine, antidiabetics, statins
**Adherence issues**	Intentionally, or unintentionally deviation from the intended usage of prescribed drugs
**Adverse effects**	Symptoms or changed laboratory values that seems to be associated with drug treatment
**Drug-interactions**	Clinically relevant drug-interactions (both drug-drug interactions and drug-supplements/herbal preparations interactions were included)
**Non-optimal drug therapy**	Adjustments in the patient's drug therapy (included both dose adjustments and temporarily stopping drug therapy) are needed due to:<br>- the acute situation<br>- reduced organ function (kidney failure, reduced liver function etc.)<br>- contraindications
**Unnecessary drug**	Drug treatment without indication according to guidelines
**Drug-related ED visit**	Considering all the above-mentioned categories, study pharmacists assessed if the current ED visit could be connected to one or more of the drugs used by the patient at admission

1. Ruths S, Viktil KK, Blix HS. [Classification of drug-related problems]. Tidsskr Nor Laegeforen 2007; 127 (23):3073-3076.
