# Supplementary material for: Impact of systematic medication review in emergency department on patients’ post-discharge outcomes—A randomized controlled clinical trial
Source: PLoS One. 2022 Sep 19;17(9):e0274907. doi: 10.1371/journal.pone.0274907 (PMC9484649; doi:10.1371/journal.pone.0274907)
Supplement: S4 Appendix — (PDF) [file pone.0274907.s004.pdf]

S4 Appendix Sensitivity analyses and survival curves

Table A. Sensitivity analysis of primary endpoint

Intention-to-treat (ITT) and per-protocol (PP) analysis of patients which died during hospital stay, had an unplanned contact with hospital (ED-visit or hospital admission) or death within 12 months after inclusion stay discharge.

	**Intervention group** ***n=400***	**Control group** ***n=397***	**P-value** [a]	**OR (95% CI)**
Patients with unplanned contact or death ITT, number (%)	214 (53.5)	217 (54.7)	0.742	0.95 (0.72, 1.26)
	**Intervention group** ***n=271***	**Control group** ***n=397***		
Patients with unplanned contact or death PP, number (%)	139 (51.3)	217 (54.7)	0.392	0.87 (0.64, 1.90)

OR: Odds ratio, CI: confidence interval

[a] P-values generated with Pearson chi^2

Table B. Sensitivity analysis on inclusion stay data.

**Inclusion stay outcomes**		**Intervention group** ***n=405***	**Control group** ***n=402***	**P-value**
Patients not hospitalized	*Number (%)*	129 (31.9%)	130 (32.3%)	0.882 [a]
Length of emergency department stay	*Median, hours (IQR, range)*	3.1 (2.1, 0.6-10.6)	3.0 (1.9, 0.6-8.9)	0.060 [b]
	Mean, hours (±SD)	3.5 (±1.5)	3.3 (±1.4)	0.086 [c]
Length of hospital stay	*Median, days (IQR, range)*	1.1 (2.6, 0.0-37.8)	1.1 (2.7, 0.0-34.1)	0.636 [b]
	Mean, days (±SD)	2.1 (±4.1)	1.8 (±2.9)	0.054 [c]

Inclusion stay endpoints presented for all patients allocated to intervention or standard care, including patients who died during hospital stay.

[a] P-values generated with Pearson chi^2

[b] P-values generated with Mann-Whitney test

[c] P-values generated with negative binomial regression

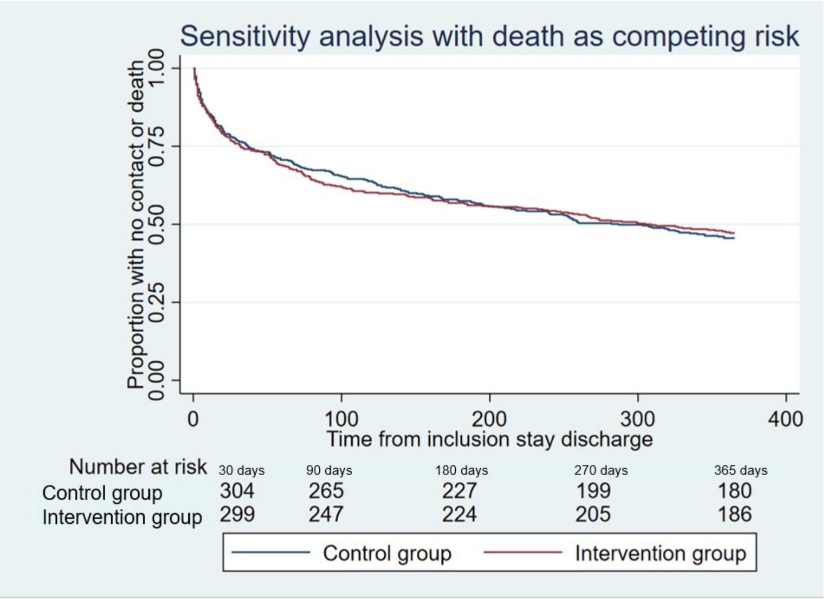

Fig A. Sensitivity *intention-to-treat* analysis with death as competing risk. All patients with follow-up data were included, intervention group n=394, control group n=395. Death during 12 months from inclusion stay discharge were treated as competing risk to unplanned contact with hospital. Median time to event (next unplanned contact with hospital or death) was 305 days for the intervention group and 287 days for the control group. This difference was not statistically significant (p=0.837, HR 0.98, 95% CI 0.81, 1.19.).

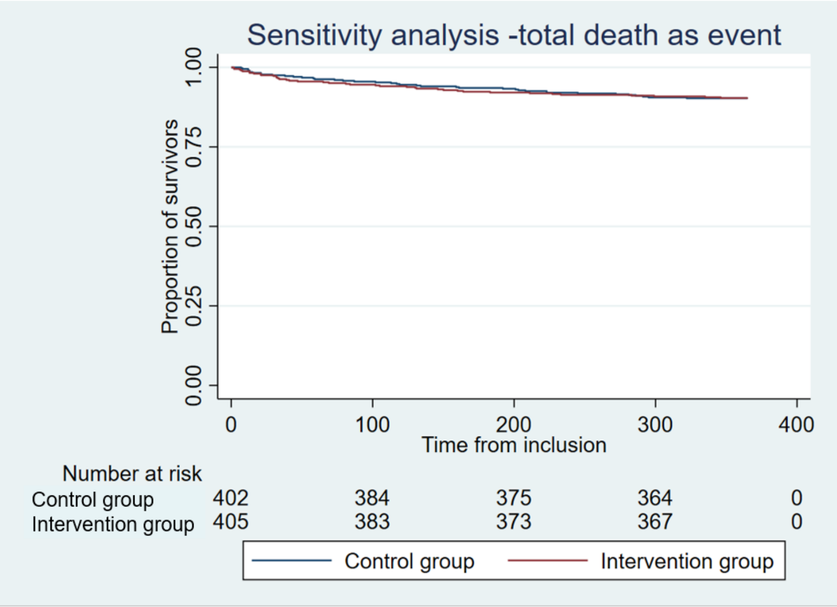

Fig B. Survival curve with death as event. All patients receiving allocated intervention or care were included. Overall death from inclusion stay admission were treated as event, i.e., both death during inclusion stay and during 12 months from hospital discharge. There was no difference between intervention- and control group regarding overall death (p=0.998, HR 1.00, 95% CI 0.64, 1.56).
