# Supplementary material for: Impact of systematic medication review in emergency department on patients’ post-discharge outcomes—A randomized controlled clinical trial
Source: PLoS One. 2022 Sep 19;17(9):e0274907. doi: 10.1371/journal.pone.0274907 (PMC9484649; doi:10.1371/journal.pone.0274907)
Supplement: Attachment — Submitted filename: PLOS One Review 2022.pdf [file pone.0274907.s005.pdf]

Dear Dr. Weinrauch and the Editorial Office of PLOS ONE:

It was my pleasure to review the manuscript entitled "Impact of systematic medication review in [the] emergency department on patients' post-discharge outcomes -a randomized controlled clinical trial" by Lisbeth Damlien Nymoen and Colleagues [PONE-D-22-04157]. The investigators presented an original investigation focusing on a systematic medication review conducted by clinical pharmacists to impact clinical outcomes and post-discharge outcomes for patients admitted to the emergency department (not to the medical wards). The investigators concluded that an emergency department pharmacist-led medication review did not significantly influence clinical- or post-discharge outcomes. The study was registered https://clinicaltrials.gov/ct2/show/NCT03123640 , and this current manuscript is the primary publication of this project.

The study design and execution were clear and documented as a quality improvement project to improve medication adherence and safety during emergency department visits. The concept was reported previously by other groups https://pubmed.ncbi.nlm.nih.gov/?term=pharmacist+led+intervention+medication+reconciliation+admission&sort=pubdate&size=200 The intervention here was not studied extensively in the past given the required resources of deploying a dedicated pharmacist to lead the medications' education and resolve discrepancies along with the complexity of operationalizing such a complex research intervention in the real world. The intervention was deployed exclusively during the initial presentation to the emergency department and not at the time of discharge. The control arm was the current standard of care without the pharmacist lead service. The included population consisted of surgical and medical patients. The exclusion was designed around the ability to engage with the patient and the priori safety measure of finding a major interaction or significant event required to notify the clinicians providing the standard of care through an interdisciplinary approach. The analysis was clear and was presented eloquently. There was a discrepancy between what the investigators stated in their clinicaltrials.gov filling and the presented primary endpoint (the wording of patient readmitted versus contact with healthcare, respectively). Though the primary endpoint was not significant, the authors took on the task of exploring their secondary outcomes. Overall, three admissions within 30 days, 90 days, six months, and 12 months were similar in the intervention and the control groups, along with other explanatory icons such as length of stay and no hospitalization.

Prior studies showed clear and reproducible efficacy of pharmacist lead effort, mainly at the time of discharge.

https://pubmed.ncbi.nlm.nih.gov/?term=pharmacist%20led%20intervention%20medication%20reconciliation%20admission%20statistically%20significant&sort=pubdate&size=200 The findings here are very interesting in the context of the global knowledge about the role of the pharmacist to improve the provided care and hence the outcomes. However, as the authors

stated, the heterogeneous population and the adaptive nature of the work could dilute the impact of the intervention.

In summation, I believe that the aim of this manuscript aligns with The Journal's scope and could add to the current knowledge and the clinical practice. Therefore, my recommendation to The Journal is to reconsider for publication after revision. Additionally, I have advised the authors regarding major and minor suggestions, which will be included in my comprehensive review and comments.

Thank you for allowing me to review this manuscript.

Sincerely yours,

Ebrahim Barkoudah, M.D., M.P.H.

TO THE AUTHORS:

The group presented an original investigation examining pharmacist-led efforts to improve medication safety during emergency department visits in this analysis. This study represents a continuation of the prior work in terms of scholarly work around the scientific concepts around medications safety within the emergency department environment. I would recommend revising the text to include a specific message, including more data from your cohort, and further refining the presented materials. Nevertheless, the aim of the manuscript should be to clarify the observation within the context of the submitted data and the specifics of the population selection and hopefully more work in the future around specific diagnosis or service line to reduce the heterogeneity of the targeted admissions. Furthermore, the conclusion should focus on extrapolating the results to positively refine the current work and positively impact the care. The presented manuscript could carry a different message regarding the importance of pharmacist-led efforts and the lessons learned for future initiatives. In addition, I have the following suggestions.

Suggestions:

- Although the rationale for this study and the primary hypothesis is stated clearly, I would recommend revising the introduction and focusing on the unique role of the pharmacist during a hospital visit, see https://pubmed.ncbi.nlm.nih.gov/?term=pharmacist+led+intervention+medication+reconciliation+admission&sort=pubdate&size=200
- Please provide the number of patients that were considered for the study before they were assessed for eligibility. Please add the number to both the results section and the figure.
- Please state clearly why hospitalization to medical or surgical wards was not chosen as the point of randomization but rather all comers to the emergency department, which could include those with high risk and low risk and a vast array of diagnoses
- Please provide the clinical characteristics of the population in both arms and compare the number of medications or categories across the two arms
- Please state the software name that was used for the analysis
- I would recommend shortening the discussion
- Please provide a statement about any possible variation of clinical management after the intervention, i.e., is there any reason for the reader to think that the patients in the intervention arm had different standards of care randomly than those in the control arm? In other words, did the intervention of pharmacist-led affect future decisions? during the emergency department stay that could lead to downstream effects of differentiation in the provided quality of care.
- Please provide more evidence for choosing your specific endpoints using the presented one; the hospitalization is a specific endpoint; however, it does not capture GP calls, admissions without emergency department visits.
